# Warthin's tumor with necrotizing tuberculous granulomatous inflammation causing severe facial nerve adhesion in parotid gland: A case report and literature review

**DOI:** 10.1097/MD.0000000000018763

**Published:** 2020-02-14

**Authors:** Shih-Lung Chen, Cheng-Cheng Hwang, Yu-Chih Liu, Wei-Ting Chen, Shih-Wei Yang

**Affiliations:** a Department of Otolaryngology & Head and Neck Surgery, Chang Gung Memorial Hospital, Linkou; b School of Medicine, Chang Gung University, Taoyuan; c Department of Pathology; d Department of Pulmonology; e Department of Otolaryngology & Head and Neck Surgery, Chang Gung Memorial Hospital, Keelung, Taiwan.

**Keywords:** facial nerve, granulomatous, *Mycobacterium tuberculosis*, parotid gland, Warthin's tumor

## Abstract

**Rationale:**

Warthin's tumor is the second most common tumor arising from the parotid gland, but it rarely occurs concomitantly with tuberculous granulomatous inflammation with only 13 documented case reports in the English literature.

**Patient concerns:**

An 82-year-old woman had a left infraauricular mass for approximately 3 years that had significantly increased in size over the previous 1 month.

**Diagnoses:**

A diagnosis of Warthin's tumor was made by ultrasonography (US)-guided core needle biopsy. Pathological examinations of the specimen obtained by total extirpation confirmed that the tumor was superimposed with tuberculous granuloma.

**Interventions:**

The core biopsy wound did not heal and there was formation of a skin fistula tract with persistent discharge. During the operation with en bloc resection of the necrotic parotid tumor, adhesion between the branches of the facial nerve was too tight to allow preservation.

**Outcomes:**

A diagnosis of necrotic Warthin's tumor superimposed with tuberculous granuloma was made. Due to the high-clinical suspicion of tuberculosis (TB) due to *Mycobacterium tuberculosis* infection, anti-TB chemotherapy was given.

**Lessons:**

Poor wound healing from a core biopsy and formation of a skin fistulous tract with persistent discharge should raise concern regarding potential extrapulmonary tuberculous infection. Although very rare, tuberculous granuloma concomitant with Warthin's tumor should be considered in the differential diagnosis of a parotid mass lesion. Adhesion of branches of the facial nerve should be expected, and sacrifice of the nerve may be planned. This consideration can be explained to the patient in preoperative counseling and planning. Anti-TB chemotherapy should be given in cases with a definite pathological report associated with speculative clinical presentation.

## 1 Introduction

Warthin's tumor (papillary cystadenoma lymphomatosum) accounts for 14% to 30% of all parotid tumors, and is the second most common tumor in the parotid gland.^[1,2]^ These lesions contain epithelial and lymphoid components.^[3]^ Parotid tuberculosis is a rare entity, accounting for only 2.8% of all parotid diseases.^[4]^ The concomitant occurrence of tuberculosis (TB) due to *Mycobacterium tuberculosis* infection within Warthin's tumor of the parotid gland is extremely rare,^[1]^ with only 13 documented case reports of TB infection within Warthin's tumor of the parotid gland in the English literature (Table 1).^[1,2,5–15]^

**Table 1:**
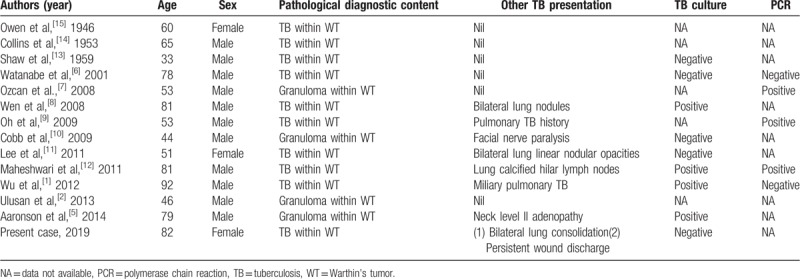
Reported cases of Warthin's tumor of the parotid gland concomitant with TB infection in the English literature.

We report a female patient presenting with a progressive enlarged mass in the left parotid gland. Poor wound healing from an ultrasonography (US)-guided core tissue biopsy occurred and caseous discharge from a formed skin tract was found. We review the literature and compared the clinicopathological characteristics of the present case with the cases reported in the English literature.

## 2 Case report

**Figure 1:**
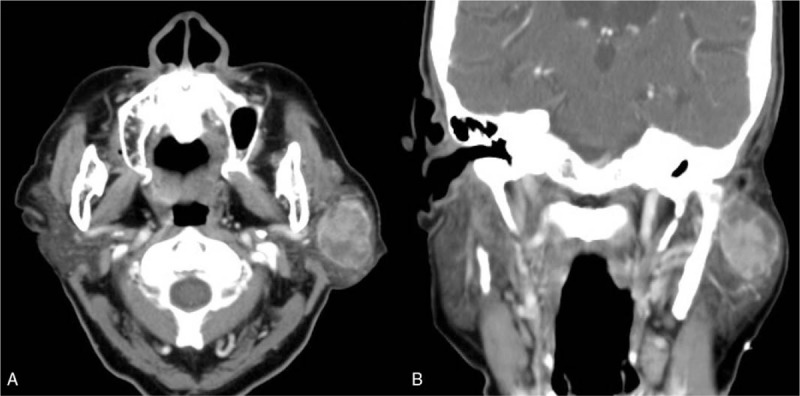
Head and neck CT scan showing a 5.5 × 3.3 cm radiopaque mass lesion with lobulated fluid accumulation crossing over the parotid gland in axial view (A) and coronal view (B). CT = computed tomography.

**Figure 2:**
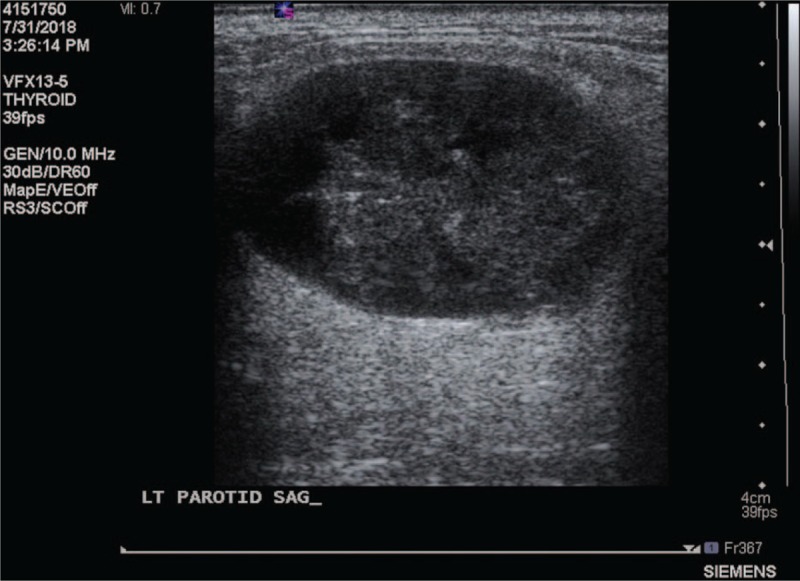
Target ultrasonography of the left parotid gland in sagittal view revealing a nonhomogeneous hypoechoic tumor with an internal hyperechoic component.

**Figure 3:**
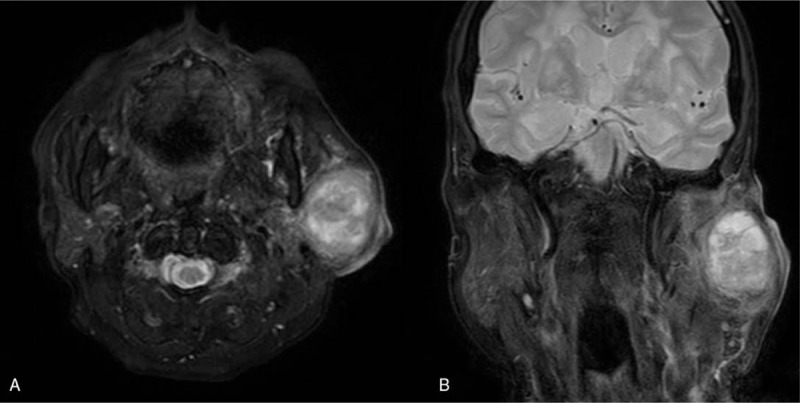
MRI revealing a necrotic mass in the superficial lobe of the left parotid gland in T2-weighted images in axial view (A) and coronal view (B). MRI = magnetic resonance imaging.

**Figure 4:**
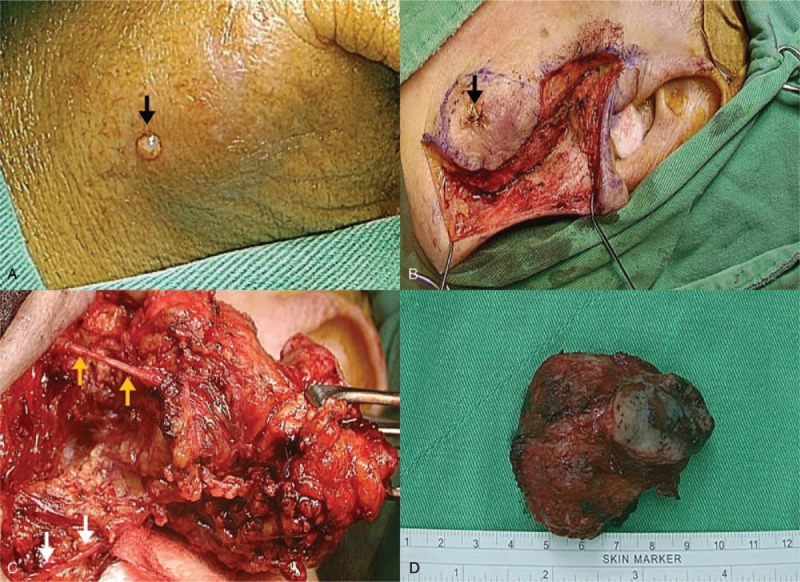
(A) Poor healing of the core biopsy wound (black arrow). A skin fistulous tract draining caseous material persisted despite surgical closure of the wound. (B) A preauricular incision was created along with a curved cervical extension following a natural skin crease below the angle of the jaw. (C) Using blunt dissection, the tumor was separated from the SCM muscle. The temporoparotid fascia was elevated and transected. The buccal (yellow arrow) and marginal mandibular branches (white arrow) of the facial nerve were clearly identified, but the soft tissue adhesion with the buccal branch was embedded in the tumor making separation of the buccal branch from the tumor mass impossible. (D) After en bloc resection, the specimen consisting of an ill-defined, yellowish, necrotic tumor measuring about 5.6 × 4.2 × 3.3 cm partially covered with skin was obtained. SCM = sternocleidomastoid.

**Figure 5:**
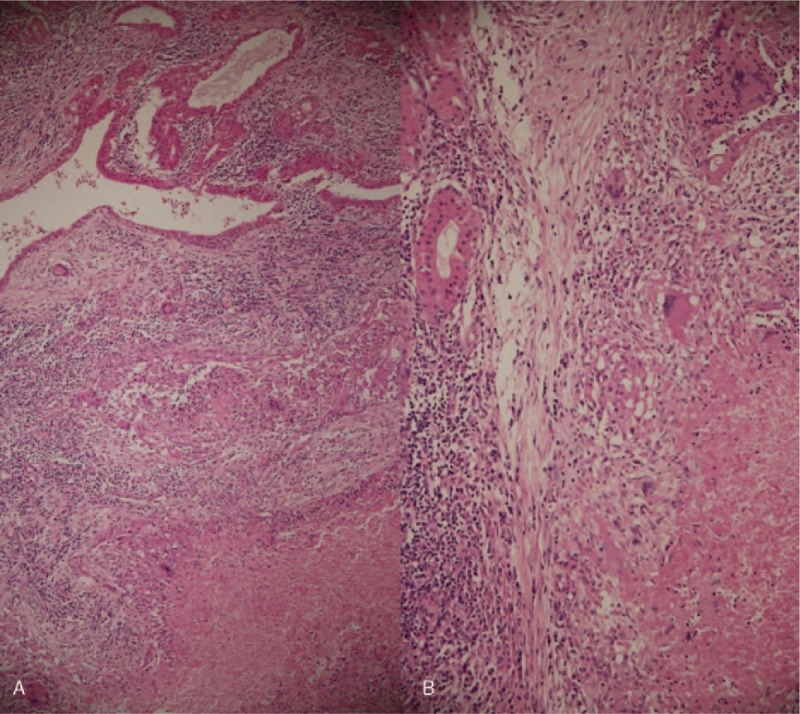
(A) Section of the left parotidectomy specimen showing Warthin's tumor. (B) An area of central necrosis surrounded by Langhans-type giant cells, epithelioid cells, and lymphocytes was seen. Residual glandular structures of Warthin's tumor composed of two-layered oncocytic and basal cells were also noted (original magnification 100×).

An 82-year-old woman came to our outpatient department with a chief complaint of a left infraauricular mass that had been slowly growing for approximately 3 years with significant enlargement over the past 1 month. The patient reported no trauma, fever, night sweating, or other discomfort related to the mass. The patient had been treated regularly for several years with bisoprolol hemifumarate and aspirin for underlying hypertension and previous stroke. Physical examination demonstrated a well-defined, fixed mass in the left parotid region without local heat, fluctuation, or regional erythema. Laboratory data revealed elevated C-reactive protein level (39.67 mg/L; normal: <5 mg/L) without leukocytosis (6500/μL; normal: 3900–10,600/μL). Head and neck computed tomography (CT) with contrast enhancement revealed an internal necrotic and lobulated fluid-accumulated mass measuring 5.5 × 3.3 cm in the superficial lobe of the left parotid gland, favoring a diagnosis of Warthin's tumor (Fig. 1A and B). Head and neck magnetic resonance imaging (MRI) revealed a necrotic mass in the superficial lobe of the left parotid gland on T2-weighted imaging compatible with Warthin's tumor (Fig. 2A and B). We performed US-guided core needle tissue biopsy for differential diagnosis of the mass lesion. Target US revealed a hypoechoic mass with some internal hyperechoic components in the left parotid gland (Fig. 3). Core needle tissue biopsy was performed and some grayish-white soft tissue was obtained. However, the biopsy site wound did not heal well and a skin fistulous tract with creamy yellowish discharge formed (Fig. 4A). Debridement of the wound and closure of the fistula with nylon sutures were performed. Nonetheless, the fistula tract did not heal and the wound still showed discharge. The pathological report of the biopsy specimen was consistent with Warthin's tumor. Total parotidectomy was performed. A preauricular incision was made along with a curved cervical extension following a natural skin crease (Fig. 4B). Using blunt dissection, the tumor was separated from the sternocleidomastoid (SCM) and digastric muscles. The temporal and zygomatic branches of the facial nerve were encased by the tumor, and severe adhesion between the nerve, peripheral parotid gland, and soft tissue hindered dissection, which made preservation of all branches impossible (Fig. 4C). After en bloc resection of the tumor, the specimen consisted of an ill-defined, yellowish, necrotic tumor measuring about 5.6 × 4.2 × 3.3 cm partially covered with the unhealed skin tract (Fig. 4D). During the postoperative course in hospital, grade IV facial nerve palsy was noted. An area of central necrosis was surrounded by Langhans-type giant cells, epithelioid cells, and lymphocytes. The residual glandular structures of Warthin's tumor were composed of two-layered oncocytic and basal cells (Fig. 5A and B). Acid-fast staining, Gomori's methenamine silver, and Periodic acid-Schiff staining were performed and all of the results were negative. Three sets of sputum cultures for pulmonary TB were negative. The pathological report confirmed Warthin's tumor with typical tuberculous granuloma. According to the pathological report and ground-glass opacity features and consolidation in the chest roentgenogram, a pulmonologist prescribed anti-TB medication (AKURIT-3, including rifampin 150 mg, isoniazid 75 mg, and ethambutol 275 mg). After discharge, the patient continued taking AKURIT-3 for 6 months. Compared to the poor healing of the core needle biopsy wound, the postoperative surgical wound healed well.

### 2.1 Ethical approval

Written informed consent was obtained from the patient for publication and any associated images. Approval of this case study was obtained from the Institutional Review Board of Chang Gung Medical Foundation (IRB No. 201900241B0).

## 3 Discussion

We report a rare case of Warthin's tumor of the parotid gland superimposed with necrotizing granulomatous infection. The postoperative parotidectomy wound healed uneventfully with adjuvant anti-TB chemotherapy. Further, we performed comparative analyses of the clinicopathological characteristics of the present case with the 13 previous case reports in the English literature.

In descending order of frequency, the extrapulmonary sites most commonly involved in TB are the lymph nodes, pleura, genitourinary tract, bones and joints, meninges, peritoneum, and pericardium.^[16]^ Extrathoracic forms of TB account for 20% of all TB cases, and TB cervical lymphadenitis is the most common form.^[2]^ TB infection in the salivary glands is rare,^[6]^ and necrotizing granulomatous infection in Warthin's tumor is an extremely rare condition.^[12,17]^ Warthin's tumor is an encapsulated cystic and solid tumor, which often presents in the tail of the parotid gland at the angle of the jaw and shows multicentricity in 10% to 20% of cases.^[2,10]^ It arises from heterotopic salivary glands within coexisting intraparotid and paraparotid lymphoid tissue.^[6,18]^ TB infection can occur within the lymphoid stroma.^[7]^

Most cases of parotid TB in Warthin's tumors in previous reports presented as a chronic, painless parotid tumor that was usually diagnosed after surgical excision. Anti-TB chemotherapy was given after the final pathology, mycobacterial cultures, or PCR results were confirmed (Table 1). It is difficult to make a definite diagnosis before operation in a patient who does not present with TB symptoms, such as night sweats, mild fever, and weight loss.^[1]^ There are two clinical forms of parotid gland TB infection, that is, a diffuse parenchymatous disease resembling a general infection^[7]^ and TB infection in the parotid gland presenting as a slowly progressing parotid mass that is difficult to distinguish from a parotid tumor.^[19]^ Although preoperative evaluations, such as US, CT, MRI, fine needle aspiration, and core needle biopsy are usually performed, definitive diagnosis is still based on the results of histopathological examinations.^[7]^ In the present case, persistent wound discharge and poor healing were noted after core biopsy despite administration of parenteral antibiotics (Fig. 4A), which has rarely been seen clinically and has not been reported previously in the literature. Facial nerve damage, hemorrhage or hematoma formation, tumor track seeding, capsule rupture, and soft tissue infection are the common complications reported to date,^[20–22]^ while there are no reports of poor wound healing in such cases. Extrapulmonary tuberculous-infected lymph nodes are usually discrete in early disease but develop into a matted nontender mass over time, and a skin fistulous tract draining caseous material may result.^[16]^ We deduced that the poor wound healing with persistent discharge was related to an active inflammatory process and suggested that bacterial and fungal cultures, including *Mycobacteria*, should be performed in such cases. Besides, intra-operative feature such as severe adhesion between the facial nerve branches and tissue of the tumor (Fig. 4C) also related to the inflammatory nature of a potential superimposed infection. The ongoing inflammation could aggravate the adhesion of the facial nerve branches and adjacent soft tissue, which should be explained to the patient during preoperative counseling and treatment planning.

Spontaneous necrosis and inflammation in Warthin's tumor have been reported in the literature.^[6]^ Eveson et al reported fibrosis, necrosis, and inflammation in 6.2% of Warthin's tumors.^[23]^ In such conditions, infarction would be the more likely culprit than infection.^[6,23]^ Spontaneous necrosis and inflammatory changes in Warthin's tumors are known as foreign body reactions when an infarction results in a rupture of the cysts and fluid containing cholesterol crystals come into contact with the stroma.^[7]^ Fine needle aspiration or core tissue biopsy may induce secondary changes with hemorrhage, fibrosis, necrosis, inflammation, and metaplastic changes due to vascular injury leading to infarction in the tumor.^[24]^ A prior needle invasive procedure accompanied with a local reactive process could be an inducing factor for the pathogenesis of granulomatous inflammatory changes, but a previous study refutes this hypothesis.^[17]^

Granulomatous inflammatory changes in salivary glands can be classified into four categories: foreign body type (reactive to X-ray contrast component), muciphagic type (ruptured retention cyst), tuberculous granulomatous change (mycobacterial infection), and sarcoid-like type (sarcoidosis, Wegener's granulomatosis, toxoplasmosis, syphilis, or idiopathic).^[17,24]^ The patient reported here underwent core needle tissue biopsy 1 month before the operation. The pathological report of the biopsy specimen revealed Warthin's tumor with glandular structures composed of two-layered oncocytic and basal cells distributed among lymphoid tissue alone. No obvious granulomatous inflammatory changes were detected. Therefore, we favored the presence of granulomatous changes in Warthin's tumor before needle biopsy and support the conclusions of Jung et al.^[17]^ The TB granulomatous inflammatory changes were confirmed in the final pathological specimen (Fig. 5A and B).

TB infection within Warthin's tumor can be confirmed by polymerase chain reaction (PCR).^[6]^ PCR assays are utilized for confirmation of TB infection in uncultured diagnosis.^[6]^ PCR can provide direct molecular detection of TB infection complex and is available for use in formalin-fixed pathological tissue specimens.^[2]^ However, multiple factors, such as repetitiveness of the amplified sequence, size of the target DNA, and the concentration of DNA should be taken into consideration when performing PCR assays.^[25]^ Tissue samples from different sites show false-negative rates of 0% to 100%.^[1]^ This may explain why several PCR results in previous reports were negative (Table 1).

As shown in Table 1, Warthin's tumor tends to occur most often in men >50 years old.^[10]^ Our patient is the third woman with parotid gland Warthin's tumor with TB infection reported to date. In addition, including our patient, the lung is the most common focus of TB infection, as shown in Table 1. A total of 6/14 cases have had such manifestations. In one case report, the patient presented with preoperative facial nerve paralysis with marginal mandibular branch involvement before surgery. Facial nerve dysfunction related to a parotid mass may happen when the nerve has been invaded by a malignant tumor. Under other circumstances, facial nerve paralysis may be associated with a rapidly enlarged benign parotid tumor, which stretches or compresses the nerve. Cobb et al considered that facial paralysis in their patient was due to displacement of the facial nerve by a rapidly expanding tumor following spontaneous infarction.^[10]^ Our patient did not show preoperative facial nerve paralysis, but during the operation severe adhesion between the facial nerve and the inflammatory tissue of the tumor made preservation impossible. To remove the tumor, the facial nerve had to be sacrificed.

Anti-TB medication is usually administered after a definite diagnosis based on pathological examination using a positive TB culture or PCR.^[6,7]^ However, certain culture methods or staining tests may not identify the bacteria,^[6,10,11]^ and PCR assays may yield false-negative results.^[1]^ By contrast, based on a complete pathological report along with clinical speculation of TB infection, a clinician can prescribe anti-TB medication as soon as possible. Therefore, we advocate that in cases with definite pathological reports and an existing speculative clinical presentation, clinicians should control TB infection in Warthin's tumor with anti-TB drugs even if TB culture is negative or PCR tests are unavailable.^[2,6,10,11]^ Such treatment can markedly improve the clinical condition of the patient.

We report a parotid mass with poor wound healing and persistent purulent discharge after a core needle biopsy, which has rarely been seen clinically. A pre-excision core needle biopsy suggested Warthin's tumor, but the surgical pathological report demonstrated Warthin's tumor superimposed with TB infection. Persistent discharge from a needle biopsy wound suggested an ongoing inflammatory process. Under such circumstances, facial nerve adhesion to the parotid tumor is possible and should raise the alertness of a surgeon. Therefore, complete dissection of the branches of the facial nerve from the tumor can be extremely difficult. Although very rare, TB infection mixed with necrotizing granulomatous inflammation within Warthin's tumor should be considered in the differential diagnosis of parotid mass lesions.

## Acknowledgments

The authors thank all of the members of Department of Otolaryngology-Head and Neck Surgery, Chang Gung Memorial Hospital, Keelung, for their invaluable help.

## Author contributions

**Data curation:** Shih-Lung Chen.

**Resources:** Yu-Chih Liu, Shih-Wei Yang.

**Supervision:** Cheng-Cheng Hwang, Yu-Chih Liu, Shih-Wei Yang.

**Validation:** Shih-Lung Chen, Cheng-Cheng Hwang, Yu-Chih Liu, Wei-Ting Chen, Shih-Wei Yang.

**Visualization:** Shih-Lung Chen.

**Writing – original draft:** Shih-Lung Chen, Shih-Wei Yang.

**Writing – review & editing:** Shih-Lung Chen, Shih-Wei Yang.

Shih-Wei Yang: 0000-0002-0979-7912.
